# Effect of Mixed Invasions of *Hypoderma bovis* and *Ostertagia ostertagi* in Cattle on Milk Yield and Contents in Polish Dairy Farms

**DOI:** 10.3390/ani11020464

**Published:** 2021-02-09

**Authors:** Agnieszka Wiszniewska-Łaszczych, Beata Wysok, Joanna Wojtacka, Marta Sołtysiuk

**Affiliations:** Departament of Veterinary Public Health, Faculty of Veterinary Medicine, University of Warmia and Mazury, Oczapowskiego 14, 10-718 Olsztyn, Poland; bea_wysok@wp.pl (B.W.); joanna.wojtacka@uwm.edu.pl (J.W.); marta.soltysiuk@uwm.edu.pl (M.S.)

**Keywords:** *Hypoderma bovis*, *Ostertagia ostertagi*, antibody, milk

## Abstract

**Simple Summary:**

The aim of this study was to determine the presence of antibodies against *Ostertagia* and *Hypoderma* in udder milk samples and the comparison of milk yield and content of the basic components of milk in ELISA-positive and ELISA-negative cows. The extensiveness of dual parasitic invasions in individual herds amounted to 3.22%, 11.36%, and 4.76% in the three studied herds. No antibodies were found in 61.2%, 22.7%, and 57.1% of the milk samples from the cows in each herd, respectively. The milk yield of ELISA-positive cows was significantly lower and amounted to 294 kg and even to 3672 kg of milk per year, per cow. No significant differences were found between the fat and protein contents in milk.

**Abstract:**

Wide distribution of ecto- and endoparasites in cattle is a serious problem in the sustainability of a farm, due to the negative impact on animals’ health and productivity. The aim of this study was to determine the presence of antibodies against *Ostertagia* and *Hypoderma* in udder milk samples and the comparison of milk yield and content of the basic components of milk in ELISA-positive and ELISA-negative cows. Milk samples were collected from 148 lactating cows from 3 herds. Antibody detection was performed using specific ELISAs for *Ostertagia ostertagi* and *Hypoderma bovis*. Milk yield and content of protein, fat, and dry matter were studied in samples from each individual cow 11 times per year at 4 week intervals. The extensiveness of dual parasitic invasions in individual herds, estimated on the basis of udder milk testing with the ELISA test, varied and amounted to 3.22%, 11.36%, and 4.76% in the three studied herds, respectively. No antibodies were found in 61.2%, 22.7%, and 57.1% of the milk samples from the cows in each herd. The milk yield of ELISA-positive cows was significantly lower in comparison to the efficiency of ELISA-negative cows and amounted to 294 kg and even to 3672 kg of milk per year, per cow. No significant differences were found between the fat and protein contents of milk between ELISA-positive and -negative cows for both parasites.

## 1. Introduction

Parasite invasions have always accompanied farm animals. A multitude of species and the wide distribution of ecto- and endoparasites in the environment is a serious problem in farm breeding due to the negative impact of the invasion on animals’ health and productivity [1].

Signs of ectoparasitic infestations are visible to the naked eye. The larvae or adults of the parasite are easy to spot on the animal’s body. The condition of the hair and skin deteriorates, and the animals become restless due to intense itching. These animals spend a lot of time fighting off insects, which translates into lower productivity on account of having less time to rest, eat, and chew [2].

Typical signs of endoparasitic infections include general weakness of the body, decrease or inhibition of weight gain. It is caused by disorders of the digestive system, malabsorption, or diarrhea. Sick animals are characterized by pale mucous membranes due to anemia. Some signs of infection by cattle parasites can be a reduction in milk production and changes in the nutritional value of milk [3,4], and/or reproductive problems, including the death of embryos and miscarriages. The severity of signs is related to the type of parasite, size of infection, food ration, environmental conditions, and the overall health of the animal. In many cases, there is a greater susceptibility to bacterial and viral infections.

The action of parasites is not always noticeable, but it can lead to chronic diseases and financial losses to the breeder.

The use of antiparasitic drugs in herds increases the losses caused by the need to introduce withdrawal periods [5] and milk disposal, which should not be used for food or animal feed. The studies described in the literature focus mainly on the identification of the invasion of one species or type of parasite and its consequences for economics and animal health [1,6]. Taking into account the specificity of dairy cattle breeding, it can be assumed that multiparasitic invasion, especially in pasture grazing cattle, may occur quite frequently and may be caused simultaneously by different species of endo- and ectoparasites.

In dairy cattle, the most common endoparasites are gastrointestinal nematodes, and among them is *Ostertagia ostertagi*—a small nematode that colonizes the abomasum of cattle and belongs to the family *Trichostrongyloidea*. Studies conducted in Germany, The Netherlands, Belgium [7], Ireland, Spain [8], and Canada [1] confirmed the prevalence of this parasite in cattle herds worldwide [9].

In turn, the most common ectoparasite in dairy herds (especially in temperate climates) is Diptera of the genus *Hypoderma*. Larval forms of the parasite cause a disease known as bovine hypodermosis. Of the six known species, the most common are *Hypoderma lineatum* and *Hypoderma bovis*. Although programs to combat hypodermosis were developed and implemented in the middle of the twentieth century in many European countries including Poland, the disease is still present and inflicts significant economic losses in breeding [10,11].

The aim of the study was to determine the extensiveness of dual parasitic invasions in individual herds based on the frequency of both anti-*Ostertagia ostertagi* and anti-*Hypoderma bovis* antibodies in udder milk and to evaluate the effect of multiparasitic invasion on the yield and content of basic milk components.

## 2. Materials and Methods

### 2.1. Selection of Herds

Taking into account the average size of a single production herd in the Warmia and Mazury voivodeship, the study was conducted in three Holstein-Friesian herds of the following sizes: heard No 1–31 heads, No 2–44, and No 3–84. All three participating herds were kept in pasture. After the end of the pasture period, a deworming program was carried out in all the herds tested. No visible signs of an existing parasitic invasion were observed in the cows.

### 2.2. Sampling

In the period from February to March 2019, 10mL of milk samples were taken from 148 lactating cows from all herds studied (No 1–31, No 2–41, No 3–76). Samples were collected according to Polish standard describing the procedure of testing milk samples (PN-A-86002: 1999) [12].

### 2.3. Determination of Antibody Titers

Milk samples were initially defatted. Antibody detection was performed using ELISA *Ostertagia ostertagi* antibody tests (Ab Svanova, Uppsala, Sweden) and a Hypodermosis Serum Screening Kit (Porquier Institute, France) according to the manufacturer’s instructions, assuming a positive result for *O. ostertagi* at OD > 0.9, while a negative result at OD < 0.4 (OD—Optical Density at 450 nm). In the case of the ELISA test detecting antibodies against *Hypoderma bovis*, a sample to positive ratio (S/P) ≥ 115% was considered as a positive result, while S/P ≤ 85% was assumed to be negative.

#### Productivity Assessment

The milk yield and content of individual components, such as protein, fat, and dry matter, were analyzed using the AT4 method in the PFHBiPM (Polish Federation of Cattle Breeders and Milk Producers) laboratory in Bydgoszcz in accordance with the decision of the EU Commission [13]. AT4 is the method developed by the Federation for the evaluation of dairy cattle. The purpose of the method is to evaluate milk yield and composition by examining udder milk samples taken from each cow on the farm at least 11 times a year at 4-week intervals. All activities related to the trial’s milking, animal identification, and the keeping of breeding documentation are performed and supervised by a certified sampler.

### 2.4. Statistical Analysis

Statistical analysis was performed with Statistica 13.0 PL software (StatSoft, Poland). The statistical analysis of the results was performed with the Kruskal–Wallis test, the results for which *p* < 0.05 was considered statistically significant.

## 3. Results

Based on the presence of antibodies in milk samples, 10 cows out of a total of 148 analyzed (6.75%) demonstrated an invasion caused by ectoparasites of the genus *Hypoderma* and endoparasites of the genus *Ostertagia*. The extensiveness of dual parasitic invasions in individual herds determined by ELISA udder milk testing varied and amounted to 3.22% in the 1st herd, 11.36% in the 2nd, and 4.76% in the 3rd herd. At the same time, no antibodies were found in 61.2%, 22.7%, and 57.1% of the milk samples from the cows in each herd, respectively. (Table 1).

A comparison of milk production in animals from all herds tested demonstrated a statistically significant (p 0.0489) decrease in milk yield in cows in which milk was positive for anti-*Ostertagia* and anti-*Hypoderma* ELISAs. The differences between the average yield of cows exhibiting positive and negative ELISA assays results in milk amounted to 294 kg in the first, 2863 kg in the second, and 3672 kg per cow in the third herd (Table 2). In the herd with the lowest decline in productivity, this value was lower than the standard deviation, while in other herds, this index showed that the least efficient cows with negative ELISA results were characterized by higher productivity than the most productive cows with positive ELISA.

The content of fat, protein, and dry matter in milk containing anti-*Ostertagia ostertagi* and anti-*Hypoderma bovis* antibodies was compared with milk without antibodies.

In the three herds analyzed, milk with antibodies was found to have a higher average of fat content (Table 3), although the observed differences were not significant (*p* > 0.05).

The protein level in the milk directly correlated with the level of dry matter. In spite of some slight changes observed in some of the herds, no significant differences (*p* > 0.05) were detected between milk with and without antibodies (Table 3).

## 4. Discussion

Following the idea of sustainable agricultural production—safe food comes from healthy animals—sustainable animal health is an essential condition for welfare. Wide distribution of ecto- and endoparasites in the environment is a serious problem in farm sustainability. Parasitic invasions are the major cause of production losses in dairy cattle herds including losses in milk production, decreased growth performance, impaired reproduction, and poor welfare [1,9,14]. Diagnosing parasitic invasions is a big challenge. The signs of parasitosis are not specific and often pass without any noticeable signs. The action of parasites leads to chronic disease and economic losses long after the invasion has ceased [9]. The development of new techniques for rapid diagnosis (ELISA tests using udder and bulk milk) allows for cheaper and more frequent monitoring of the situations related to the occurrence of parasitic invasions. Antibodies persist in the body for a long time, therefore many of the alarming symptoms and decline in animal productivity caused by parasites may be explained [15,16]. Despite the high awareness of farmers and milk producers, reluctance to use antiparasitic drugs is observed, which is related to the necessity of introducing withdrawal periods and milk disposal.

The studies conducted so far, as indicated by published data, focused on the identification of the invasion of one species of parasite and the resulting losses. Studies carried out in various countries show that the average extensiveness of gastrointestinal nematode infection (endoparasites) ranges from 61.96% in Mexico [17], 56% in Canada [18] to as much as 90% in Belgium and The Netherlands [19]. Polish studies, limited to the Warmia and Mazury voivodeship, showed extensiveness of infection at the level of 20% [20].

Similar observations were made with respect to the infestation of parasites of the genus *Hypoderma*. At the end of the twentieth century, in many European countries including Poland, a program of hypodermosis control was introduced that significantly reduced parasite occurrence in production herds [10]. However, as shown by later studies conducted by employees of the State Veterinary Institute in Pulawy, the extensiveness of infestation in Poland after the end of the program again reached the level of 20% to 100% of the population of cattle herds [11]. The studies carried out in the Warmia and Mazury voivodeship showed extensiveness of infestation at the level of 28% [21].

Taking into account the environmental conditions, the farming method, and the general rules of the ecosystem, it can be concluded that multiparasitic invasions are not rare. There are few studies on the incidence of multiparasitic diseases in dairy cows and their impact on cows’ productivity. An extensive study on the occurrence of endoparasites in cattle was carried out in Romania. The results obtained by Chihai [22] show that as much as 69.5% of the cattle were colonized by two or more species of parasites, 24.5% by one species, and only 6.5% of cows were free from the invasion. The current study presents the extensiveness of dual parasitic invasion, as assessed by ELISA testing of milk samples, in three herds in the Warmia and Mazury voivodeship that was determined at the level of 3.2% to 11.3% of the herd populations.

A major problem associated with parasitic invasion in dairy cattle herds is the economic losses due to reduced productivity. The decline in milk production in herds invaded by the gastrointestinal nematode was estimated to be on average between 327 and 436 kg of milk/year in Belgium, France, Spain, and Nova Scotia [7,19,23,24]. Studies carried out in northeastern Poland showed losses of 863 kg/year [19]. The decrease in milk production in herds with confirmed infestation of *Hypoderma* spp. was estimated at 681.8 kg/year in the Warmia and Mazury voivodeship [20]. In the case of ectoparasites, losses associated with skin damage as well as changes in meat due to migration of larvae cannot be overlooked. The reduced value of cattle for slaughter, associated with the infestation of *Hypoderma* spp., was widely described in the literature, and in some countries, annual losses are estimated at hundreds of thousands of euros [2,10,25].

As demonstrated by the results presented in the study, comparing the milk yield of dairy cows that did not show the presence of antibodies in milk (ELISA-negative) with the milk yield of ELISA-positive cows, there was a statistically significant decrease in milk production, even reaching 53% per cow. Changes in the content of basic milk components were also observed; however, these differences were not statistically significant. The most likely cause of the high fat content is parasite-induced ketosis—in order to achieve balance, the cow’s body excretes ketones as fat in the milk [26]. The percentage of protein was closely correlated with the amount of dry matter in milk.

These economic losses are, however, not only associated with reduced milk yield but the costs incurred by farmers associated with parasitic invasions in the herd also include economic losses caused by the reduced slaughter value of these animals. Loss estimates should take into account activities related to antiparasitic prevention, and consequently, the costs of retaining withdrawal periods for milk and meat in the case of using antiparasitic preparations [1,9,14].

## 5. Conclusions

The results obtained in the research indicate that dairy cattle grazing in a pasture system are exposed to multiparasitic invasions, which reduce milk productivity.

The availability of diagnostic tests enabling the rapid detection of antibodies in milk samples creates new opportunities for parasitological control in the herd. The awareness of the presence of mixed invasion can contribute to the development and implementation of new prevention programs aimed at comprehensive protection of herds against various groups of parasites. Therefore, a wide range of prophylaxis should be applied on farms, consisting of selecting a deworming program, reducing the contamination of pastures and livestock housing with invasive forms. The risk of invasion can be reduced by using quarters grazing and taking care of the hygiene of cowsheds and paddocks, increasing, in this way, production yields.

## Figures and Tables

**Table 1:** Extensiveness of dual parasitic invasions determined by ELISA udder milk testing in the herd tested.

Herd	The Size of the Herd	Milk ELISA *Hypoderma*/*Ostertagia*	N	%
No1	31	+/+	1	3.22
		−/−	19	41.2
No2	44	+/+	5	11.36
		−/−	10	22.7
No3	84	+/+	4	4.76
		−/−	48	57.1

**Table 2:** The numerical values of milk yield (mean and STDM *—standard deviation of the mean).

Herd	Milk ELISA *Hypoderma*/*Ostertagia*	Milk Yield [kg]	
		Annual Average	STDM *
No1	+/+	5984	0
	−/−	6278	644
No2	+/+	3649	536
	−/−	7771	1099
No3	+/+	4908	1339
	−/−	8580	404

**Table 3:** The numerical values of basic components of milk (mean and STDM *—standard deviation of the mean).

Herd	Milk ELISA *Hypoderma*/*Ostertagia*	Fat [%]		Protein [%]		Dry Matter [%]	
		Annual Average	STDM *	Annual Average	STDM *	Annual Average	STDM *
No1	+/+	5.16	0	3.73	0	14.4	0
	−/−	4.69	0.13	3.22	0.066	13.3	0.17
No2	+/+	4.99	0.13	3.10	0.068	13.52	0.15
	−/−	4.06	0.18	3.32	0.076	12.87	0.23
No3	+/+	4.53	0.089	3.03	0.19	13.01	0.20
	−/−	4.43	0.066	3.35	0.039	13.32	0.095

## Data Availability

The analyzed data is presented in this study.
